# Comparative Evaluation of Surface Roughness of Porcelain Fused to Metal and Stainless-Steel Crown Following Ultrasonic and Hand Scaling: A Comparative Study

**DOI:** 10.7759/cureus.27134

**Published:** 2022-07-22

**Authors:** Hiroj Bagde, Nikhat Fatima, Pragati Sharma, Anant Ragav Sharma, Gaurav Agrawal, Pooja Agrawal

**Affiliations:** 1 Periodontology, Rama Dental College and Research Centre, Kanpur, IND; 2 Prosthodontics, Bhagwatii Dental Care Hospital, Gorakhpur, IND; 3 Periodontics, Pacific Dental College, Udaipur, IND; 4 Prosthodontics and Crown and Bridge, New Horizon Dental College and Research Institute, Bilaspur, IND

**Keywords:** profilometry, sem, prosthetic crowns, srp, surface roughness

## Abstract

Introduction

Surface roughness encourages plaque retention and causes mechanical, chemical, and biological irritation to surrounding soft tissues. Hence, a smooth surface of restoration is preferred for optimal plaque control and the health of the periodontium.

Aim

The aim is to evaluate and compare the surface roughness of porcelain fused to metal and stainless-steel crowns following ultrasonic and hand scaling techniques.

Material and methods

An in-vitro study was conducted on 30 porcelain fused to metal crowns and 30 stainless-steel crowns. Their surface roughness following instrumentation was evaluated by scanning electron microscope (SEM) and profilometry.

Results

Evaluation by profilometry indicated that porcelain fused to metal following ultrasonic instrumentation has a statistically more significant surface roughness and indentation as compared to hand scaling with p-values < 0.05.

Conclusion

The surface roughness of any restoration may act as a plaque retentive factor which would affect the health of the periodontium. Ultrasonic scaling is capable of creating roughness to a more extent as compared to hand scaling and porcelain fused to the metal type of restorations is more vulnerable to roughness.

## Introduction

Oral health is defined as freedom from physical disease or pain [1]. The primary concern of a dentist is to bestow a disease-free oral environment and to provide good oral functionality in the form of speech, mastication, etc. Periodontal care is of utmost importance considering the multifaceted perspectives of oral health. Surface roughness has its own potential to cause irritation and plaque retention in the stainless-steel crown as well as porcelain fused to metal (PFM) crowns. The goal of the study is to look at the problems and factors that cause plaque to stick around, as well as to compare the different scaling methods.

The oral cavity is of paramount value considering two factors: 1) the attachment of teeth to the bone tissues of the jaw and 2) the integrity of the surfaces of the masticatory mucosa. The periodontium is called the attachment apparatus or the supporting tissue of teeth. It constitutes a developmental, biological, and functional unit that undergoes certain changes with age. In addition to this, it is subjected to morphological changes related to functional alterations and alterations in the oral environment [2]. Since the 1960s, it has been clear that there is a link between dental plaque and problems with the gums. professionals became aware of the importance of plaque control. Consequently, patients started to get involved in daily personal oral hygiene, which included both mechanical and chemical plaque control measures. The relationship between denture plaque, oral mucosa, and diseases have been extensively studied and widely acknowledged by dentists. Therefore, prosthesis wearers have been well instructed on the importance of its cleanliness for the maintenance of oral health. A glazed surface of a prosthesis would make the surface smoother by decreasing the accumulation of residual food, minimizing plaque adhesion, and improving oral hygiene conditions [3]. However, the roughness of the surface has been reported to abrade adjacent and opposing teeth, which encourages plaque retention. It mechanically irritates surrounding soft tissues [4]. So, a strict maintenance plan can make sure the prosthesis lasts as long as possible by keeping the soft tissues in good shape [5]. The goal of this study is to use scanning electron microscopy and profilometric analysis to figure out how rough the surfaces of different fixed prosthetic materials are after periodontal scaling.

## Materials and methods

**Figure 1:**
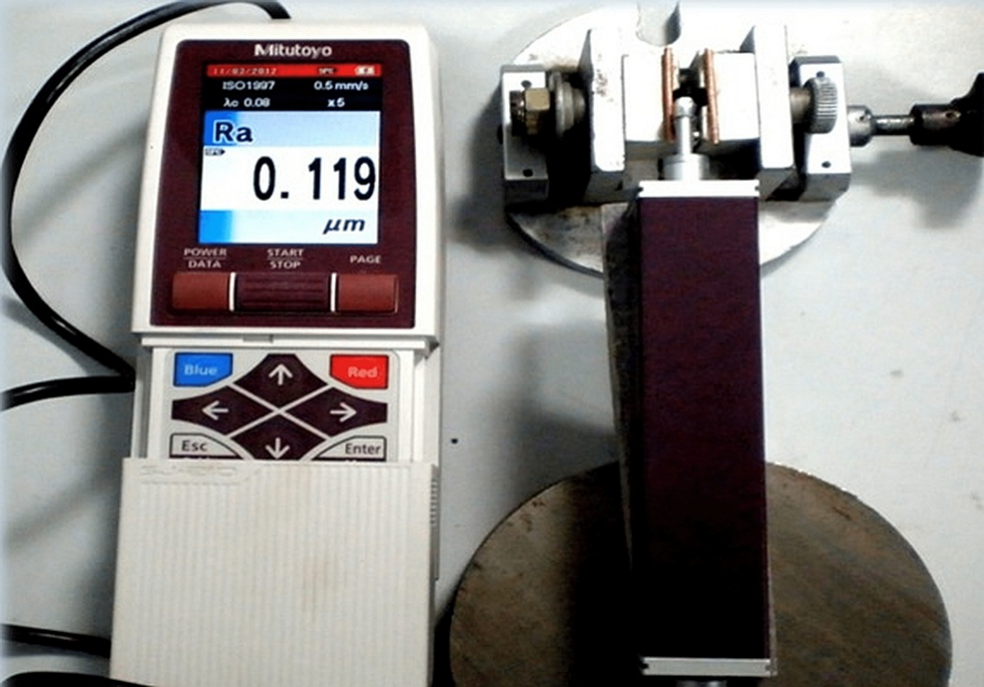
Profilometric machine

Thirty porcelain fused to metal (PFM) and 30 metal crowns were included in the study. They were vacuum-fired and glazed by standard procedures in a porcelain furnace. As a result of this, uniform porcelain and a metal surface were achieved for the samples. The surface roughness of both metal and PFM glazed surfaces was studied by the use of a profilometer machine (Figure 1) with 0.1 µm accuracy.

For both surfaces, indentations were evaluated prior to instrumentation. In addition to this, SEM photographs were taken of the glazed surfaces of metal crowns and PFM crowns before instrumentation with an SEM microscope.

Scaling was performed on the buccal cervical aspects of the crowns mounted on the casts. Thirty samples of metal crowns and 30 samples of PFM crowns were scaled by periodontal hand- instrumentation using a posterior Jacquette scaler and by ultrasonic scaler buccal cervical aspect. The duration for scaling was fixed at 1 minute for each sample. The hand instrumentation was carried out on the surface for 1 minute by use of a posterior Jacquette scaler beveled at 45-degree angulation with oblique strokes. For the ultrasonic instrumentation, the surface scaling was performed by the EMS scaling unit at medium speed. The scalar tip was used for 1 minute on the buccal cervical surface with an angulation of 0-15 degrees with oblique strokes. SEM photographs were taken after instrumentation (hand and ultrasonic) for both metal and PFM crowns so as to compare the surface indentations for the instrumentation techniques with the help of an SEM machine.

Materials are important to highlight the importance of the tools and techniques to justify the process of implementing the technology for plaque removal. It has utilized modified and efficient tools to understand the definite point of differentiation between the scaling methods. The purpose of the study is to understand the presence of plaque within the porcelain fused metal after the hand scaling and the way ultrasonic scaling has removed the plaques. It might be purposeful to understand the definite activities to make the process accelerate and fuse the methanol to reduce the chances of roughness.

A profilometric machine is important to read and count the measurement of roughness which helps to make the scaling process with proper scaling methods. It may earn the strategy to make the process easier and more authentic so that each of the sections of the crown can be analyzed for its roughness prior to and after instrumentation. Stainless-steel crown has a positive side as the steel used is there to reduce the mechanical and physical roughness [1]. Technical studies have suggested implementing ultrasonic scaling to remove the process of plaque within the metal crown. It might give the product sustainability. So also in the case of PFM the forces exerted by the scaler tips and hand instruments can cause the porcelain to chip off and cause exposure of the crown as well as plaque retention due to the created roughness.

Scanning electron microscope is another microscope system that can in-depth analyze the surface texture of the used crowns and then to determine and correlate the roughness created by various procedures [2]. To reduce any bias, the instrumentation procedure was performed by the same operator. The mean value of the surface roughness was calculated on both PFM and metal surfaces before and after hand and ultrasonic instrumentation. Ra value is the mean of the surface roughness calculated by the profilometer when it is in contact with the crown surface whereas Rz is the mean of the peak to valley height when seen on the profilometer reading and calculated by the profilometer itself. It was intended to correlate the surface roughness values for different materials and to compare both instrumentation techniques.

## Results

The study evaluated the surface roughness with the use of a profilometer. All the crowns were taken to analyze the relative depth of the roughness, which was then measured and analyzed for any statistical significance. The mean surface roughness (Ra), and mean peak to valley height (Rz) values for both the crowns before and after the instrumentation were obtained as displayed in Tables 1, 2.

**Table 1:** Mean roughness value and Mean peak to valley height (Ra and Rz) on metal crowns in µm. Ra - average roughness, Rz - maximum roughness, µm - micro-meter

	Ra	Rz
No Scaling	0.047	0.229
Hand scaling	0.069	0.343
Ultrasonic scaling	0.119	0.530

**Table 2:** Mean roughness value and Mean peak to valley height (Ra and Rz) on porcelain fused to metal crowns in µm. Ra - average roughness, Rz - maximum roughness, µm - micro-meter

	Ra	Rz
No scaling	0.067	0.319
Hand scaling	0.144	0.696
Ultrasonic scaling	0.288	1.965

Table 1 has described the roughness value with Ra and the mean peak valley height with Rz, where it has defined the strategy to understand the value. The table has implemented the comparison between no scaling, hand scaling, and ultrasonic scaling with a definite course of mitigating the roughness of the ceramic metal. No scaling has the value of Ra at 0.047 m and Rz at 0.229 m, whereas hand scaling has shown Ra at 0.069 m and Rz at 0.343 m. Ultrasonic scaling has the ability to understand the mean value of metal crowning for Ra of 0.119 m and Rz of 0.530 m.

Table 2 has defined the porcelain fused to the metal crown through the roughness created through scaling, hand scaling, and ultrasonic scaling as per the preferences. No scaling method has shown the value of Ra up to 0.067 m and Rz up to 0.319 m. On the contrary, hand scaling has defined the value of 0.144 m for Ra and 0.696 m for Rz. The ultrasonic system has defined the core value with 0.288 m for Ra and 1.965 m for the Rz with the mean tendency nearest to the actual value. By explaining how the table analysis works, one could come to the conclusion that ultrasonic scaling can make PFM restorations more rough.

The result of the two-way ANOVA test for Ra and Rz values for the groups revealed significant difference at p-value < 0.05 for both the crowns as well as for both the instrumentation techniques. Table 3 has defined the descriptive statistics for Ra, which could understand the point of significant differences between two methodologies. The table has narrated the p-value for the hand scaling up to 0.009 and for the ultrasonic up to 0.001, which shows the p-value < 0.05 for both of the scaling methods. This value shows the significant differences between these two procedures to understand the necessity of implying the proper method for stainless steel crowns. Thus, hand scaling has created more roughness on PFM crowns as compared to metal crowns so is the same case with ultrasonic scaling.

**Table 3:** Descriptive statistics for Ra Ra - average roughness

Scaling type	Metal crowns	Porcelain fused to metal crown	P-value
Hand scaling	0.0686 ± 0.007	0.134 ± 0.0283	0.009
Ultrasonic scaling	0.0983 ± 0.018	0.304 ± 0.06	0.001

Table 4 has justified the descriptive statistics for Rz, which could have the tendency to imply two different values for hand scaling and ultrasonic scaling. The hand scaling of Rz has defined the p-value for hand scaling as 0.025 and ultrasonic scaling 0.009 to define the tendency to show the unlikeness.

**Table 4:** Descriptive statistics for Rz Rz - maximum roughness

Scaling type	Metal crowns	Porcelain fused to metal crown	P-value
Hand scaling	0.476 ± 0.274	0.621 ± 0.24	0.025
Ultrasonic scaling	0.461 ± 0.082	2.014 ± 0.136	0.009

**Figure 2:**
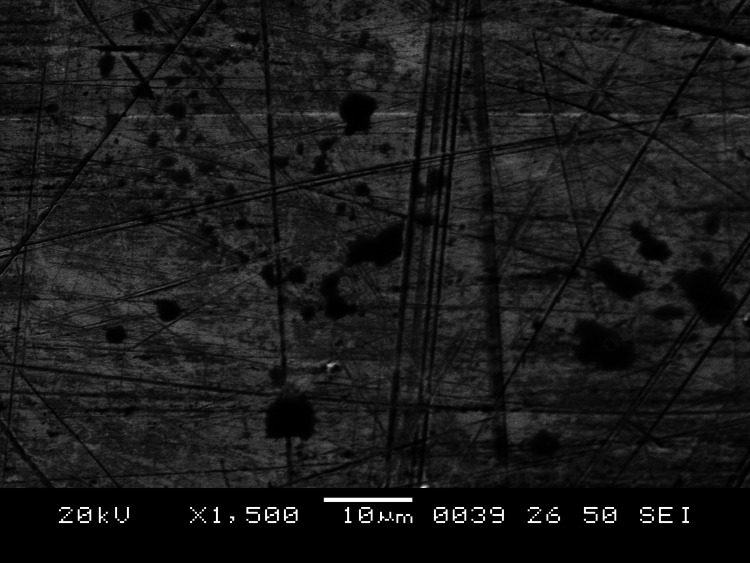
SEM image showing roughness created SEM - scanning electron microscope

Following hand, instrumentation mean surface roughness and maximum peak to valley height was higher for PFM crowns. So also subsequent to ultrasonic instrumentation mean surface roughness was significantly higher on PFM crowns as compared to hand instrumentation. This was proved by profilometry and SEM photographs (Figure 2). The various parameters were also corelated with the SEM images.

## Discussion

A healthy periodontium is critical for the longevity of the tooth and prosthesis. Because plaque is the primary cause of gingivitis and periodontitis [6,7], optimal plaque removal plays a very important role in the longevity of any prosthesis. Mechanical and chemical plaque removal methods like tooth brushing, interdental aids, and mouth rinses can be carried out on a daily basis by patients at a personal level. At the professional level, a dentist performs scaling either manually or with the aid of powered ultrasonic scalers. As a result of professional scaling, micro-abrasions are formed on the root surface. Since this micro-abrasion can act as a plaque retentive factor, polishing is done in order to obtain a smooth surface [7]. Scaling is accomplished by scaling. It can also cause abrasion on the cervical one-third of the crown. These micro-abrasions accumulate and retain plaque. After several days of undisturbed plaque, more mature plaque formation occurs at the sites. It leads to constant microbiological and chemical irritation of the surrounding tissues. This, in turn, makes the areas around the prosthesis more prone to inflammation and bleeding [8].

When the two surfaces were compared, statistically significant surface roughness was seen for the ultrasonic instrumentation. No reference in the literature is available to support this result. However, Lee in a study evaluated the effects of ultrasonic scaling and periodontal curettage on the surface roughness of PFM crowns. He arrived at a conclusion that PFM crowns following ultrasonic scaling in an SEM analysis have higher surface roughness [9]. The mean peak to valley height, i.e., indentation value in the intergroup and intragroup showed that ultrasonic instrumentation in porcelain fused to the metal crown has the highest indentation, i.e., Rz value. An in-vitro study done by Zortuk et al. demonstrates that the mean surface roughness (Ra values) greater than 0.2 m shows increased bacterial adhesion of *P. gingivalis* [10]. In a comparative evaluation done on the root surfaces by hand and ultrasonic instrumentation, Renato et al. observed significant root surface defects. He opines that these defects act as a plaque retentive factor [11]. In a recent study by Fox et al., the surface roughness was evaluated on the titanium implant surfaces by plastic and metal curettes. As a consequence, significantly higher surface roughness was found in relation to metal curette [12]. Thus, surface roughness, i.e., the Ra and Rz values, is responsible for plaque retention and the health of the periodontium. This finally affects the longevity of the prosthesis [13,14].

Both methods are able to cause surface roughness, but ultrasonic scaling due to its aggressive nature of movement has been responsible for higher surface roughness values for both types of crowns [15]. However, as the limitations of the study suggest, an in vivo study with a bigger sample size incorporating various other restorative materials could be a future prospect.

## Conclusions

Surface roughness has a significant impact on the Ra values, which in turn affects the plaque retention of the restorative material. So, the longevity of the prosthesis and the health of the periodontium are influenced. The present study has defined the comparative evaluation of surface roughness of PFM and stainless-steel crowns following ultrasonic and hand scaling. The initial stage of the study has defined the statistical analysis through the methods of justifying the comparison. It has put up the process of defining the p-value or value for assessing the differentiation between no scaling, hand scaling, and ultrasonic scaling. It has helped to understand the necessity of plaque removing the actual and suitable process with value calculation and comparison. This method has defined the narration of actual materials and methods for counting the roughness and has utilized the electron kinetic energy for the valent orbit attraction after the corrosion.
